# Non-Isocyanate Polyurethane Bio-Foam with Inherent Heat and Fire Resistance

**DOI:** 10.3390/polym14225019

**Published:** 2022-11-19

**Authors:** Dallin L. Smith, Danixa Rodriguez-Melendez, Sidney M. Cotton, Yufeng Quan, Qingsheng Wang, Jaime C. Grunlan

**Affiliations:** 1 Department of Chemistry, Texas A&M University, College Station, TX 77843, USA; 2 Department of Chemical Engineering, Texas A&M University, College Station, TX 77843, USA; 3 Department of Mechanical Engineering, Texas A&M University, College Station, TX 77843, USA; 4 Department of Materials Science and Engineering, Texas A&M University, College Station, TX 77843, USA

**Keywords:** rigid foam, non-isocyanate polyurethane, tannic acid, chitosan

## Abstract

Polyurethanes (PUs) are versatile and widespread, particularly as flexible and rigid foams. To avoid isocyanates and other toxic reagents required for synthesis, such as phosgene, alternative synthetic routes have been utilized to produce non-isocyanate polyurethanes (NIPUs). A thermally and flame-resistant rigid NIPU was produced from environmentally benign and bio-sourced ingredients, requiring no catalyst or solvents. A foamed structure was obtained by the addition of glutaraldehyde and four different carboxylic acids: malic acid, maleic acid, citric acid, and aconitic acid. The resulting morphology, thermal degradation, and flame resistance of each foam were compared. The properties vary with each carboxylic acid used, but in each case, peak thermal degradation and peak heat release are postponed by >100 °C compared to commercial rigid PU foam. Furthermore, in a butane torch test, NIPU foams exhibit an 80% higher remaining mass and a 75% reduction in afterburn time, compared to commercial polyurethane. This bio-based polyurethane eliminates the hazards of traditional PUs, while imparting inherent thermal stability and flame resistance uncharacteristic of conventional foams.

## 1. Introduction

Polyurethanes (PU) are a versatile class of polymer that have been utilized in numerous industries since their discovery by Otto Bayer almost a century ago. They combine durability, toughness, and flexibility in a way few other materials do, which makes them well-suited for upholstery, packaging, coatings, and even biomedical devices [1]. Additionally, PU properties can be modified through intentional selection of reagents. Currently, almost two-thirds of polyurethane production is for rigid and flexible foams, with the remainder being used in nonporous binders, adhesives, and coatings [2]. Most rigid foam is used in construction and insulation, whereas flexible foam is common in furniture and footwear.

Traditionally, polyurethanes are formed by reacting isocyanates with polyols to form a polymer of repeating carbamate linkages. The properties of the final polymer can be controlled by careful selection of starting materials. Polyethers and polyesters are the most common polyols, but polycarbonates, polyacrylates, or hydroxyl-terminated polybutadienes are also feasible, given they contain an average hydroxyl functionality of two or more [3]. Isocyanates can be aliphatic (hexamethylene diisocyanate, isophorone diisocyanate, methyl isocyanate) or aromatic (methylene diphenyl diisocyanate, 1,5-napthylene diisocyanate, toluene diisocyanate). Unfortunately, isocyanates are hazardous at all stages of their production and use. For example, toxic phosgene gas is typically used to convert amines to isocyanates [4]. Furthermore, isocyanates themselves are damaging to mucous membranes and skin [5,6]. Some are known animal carcinogens and suspected human carcinogens [7]. During combustion, PUs release isocyanates, which degrade into HCN, another dangerous gas. Moreover, at the end of their lifetime, landfilled polyurethanes produce toxic amines during hydrolysis [8].

Due to the hazards associated with polyurethanes, there is a growing effort to produce them without isocyanates. These so-called non-isocyanate polyurethanes (NIPUs) rely on alternate reactions to produce carbamate moieties with different constituents [8,9,10]. For example, the rearrangement of acyl azides and their subsequent condensation with alcohols results in polyurethanes, but it still generates isocyanates in situ [11]. Some methods avoid isocyanates altogether, but their precursors still require phosgene or similarly toxic reagents (e.g., polycondensation between chloroformates and amines or carbamates and alcohols) [8]. As early as the 1950s, organic carbonates and polyamines have been investigated as safer alternatives to phosgene derivatives and isocyanates and contemporary work continues [12,13,14,15,16,17,18,19]. These reactions require heating or a catalyst in order to proceed at practical rates, but they are widely considered the most promising alternatives.

As with traditional PUs, NIPUs can be produced as a foam by utilizing a blowing agent. For example, siloxanes can react with amines to release hydrogen gas [16,20,21,22]. Alternatively, carboxylic acids and sodium bicarbonate have been used to foam in conjunction with hardeners [23,24,25,26]. Although the formation of the polyurethane requires heating or a catalyst, foaming can easily take place at ambient temperature. In many cases, however, NIPU foams exhibit poor thermal or fire resistance, as do most commercial polyurethanes [20,21,22,23,24]. The ability to create NIPU foam with thermal and flame resistance will accelerate their replacement of traditional polyurethanes. Whereas typical PU reagents are limited in their properties, NIPU starting components that contribute thermal or fire resistance can be utilized. For example, Chen et al. observed increased LOI values by increasing condensed tannin content in a glucose-based NIPU foam [26]. Likewise, Sternberg and Pilla utilized lignin to produce a NIPU foam with superior thermal stability [16].

In the present study, chitosan (a natural polysaccharide) is incorporated into a NIPU with tannic acid (a natural polyphenol). These two bio-sourced ingredients have been shown to facilitate charring and improve flame resistance in other systems including PU foam [27,28,29,30]. Other tannins have also been successfully incorporated into non-isocyanate polyurethanes [22,31]. As dimethyl carbonate is considered a green reagent [32], the present method not only lacks harsh chemicals or solvents but also imparts inherent flame resistance. These advantages demonstrate the Principles of Green Chemistry 3, 4, 5, 7, and 12. Additionally, self-blowing is performed at room temperature without a catalyst to yield a rigid foam. Four carboxylic acids were compared as the blowing agent in this recipe. Citric acid contains three −COOH groups and an alcohol, whereas malic acid contains two. Aconitic acid and maleic acid are the unsaturated counterparts to citric acid and malic acid, respectively. The resulting foams self-extinguish immediately following a 10-s exposure to a butane torch flame. In addition, the principal weight loss or heat release event in TGA or MCC is delayed by >100 °C compared to traditional rigid polyurethane foam. This NIPU requires no further treatments or additives and offers superior thermal performance to commercial rigid foam. Variations of this material could potentially be utilized as insulation in construction.

## 2. Materials and Methods

### 2.1. Materials

Dimethyl carbonate (99%, DMC), tannic acid (TA), glutaraldehyde (50 wt.% in water, GA), citric acid monohydrate (≥98%, CA), trans-aconitic acid (98%), DL-malic acid (≥99%), maleic acid (≥99%), and DMSO-d_6_ (99.5%) were purchased from MilliporeSigma (Burlington, MA). Chitosan (95% deacetylated, CH) was purchased from Greentech Biochemicals (Qingdao, China). Hexamethylenediamine was purchased from VWR (Radnor, PA). All water was deionized (18 MΩ). Rigid commercial polyurethane foam was purchased from Dick Blick Art Materials (Sculpture Block, Galesburg, IL, USA).

### 2.2. Resin Synthesis and Foaming

The NIPU resin was prepared based on a procedure adapted from Chen et al. [25]. First, 10.0 g tannic acid, 10.0 g chitosan, 33.3 g deionized water, and 27.0 g dimethyl carbonate were added to a round bottom flask fitted to a reflux condenser with a magnetic stirrer. The mixture was stirred and heated to 65 °C for one hour. Next, 77.6 g of hexamethylenediamine (70 wt.% water solution) was added, stirred, and heated to 90 °C for two hours. The NIPU resin was then cooled to room temperature and homogenized before use. The ambient foaming of the resin was performed by quickly adding 5 g of blowing agent solution to 5 g of NIPU resin and immediately stirring for a few seconds. Blowing agent solution consists of carboxylic acid (50 wt.% in water) and glutaraldehyde (50 wt.% in water), for a molar ratio of approximately 1:2 (acid: glutaraldehyde). Once mixed, the foams were placed in a desiccator for 72 h to remove excess moisture. Samples were equilibrated under ambient conditions prior to testing (68–80% relative humidity, 20–23 °C).

### 2.3. Characterization

Fourier transform infrared spectroscopy (FTIR) was performed with a Bruker Alpha Platinum ATR−FTIR spectrometer (Billerica, MA, USA). Chemical structure was analyzed with ^1^H NMR spectroscopy (Inova 500 MHz spectrometer operating in the FT mode with DMSO-d_6_ as the standard). Foam structure was evaluated using images from a field emission scanning electron microscope (FE-SEM), with a 5 nm thick Pt/Pd alloy applied by sputter-coating (Model JSM-7500, JEOL, Tokyo, Japan). The thermal degradation of samples was observed under a 60 mL∙min^−1^ sample flow of air and 40 mL∙min^−1^ balance flow of nitrogen, with a TA Instruments TGA Q50 (New Castle, DE, USA). Samples were held at 100 °C for 20 min to remove excess water and heated at a rate of 10 °C∙min^−1^ to 700 °C. Microscale combustion calorimetry was performed with a ramp rate of 1 °C∙s^−1^ (Fire Testing Technology, East Grinstead, UK). Between 3−4 mg of samples were heated under a flow rate of 80 cm^3^∙min^−1^ nitrogen and the thermal degradation products were mixed with a 20 cm^3^∙min^−1^ stream of oxygen before entering a 900 °C combustion furnace. Foam flammability was evaluated using a butane torch with a 2.5 cm inner blue flame (Bernzomatic ST2200T, Worthington Industries, Columbus, OH, USA). A 1 cm^3^ cube was placed 5 cm from the torch and held in the flame for 10 s.

## 3. Results and Discussion

### 3.1. NIPU Synthesis

**Figure 1:**
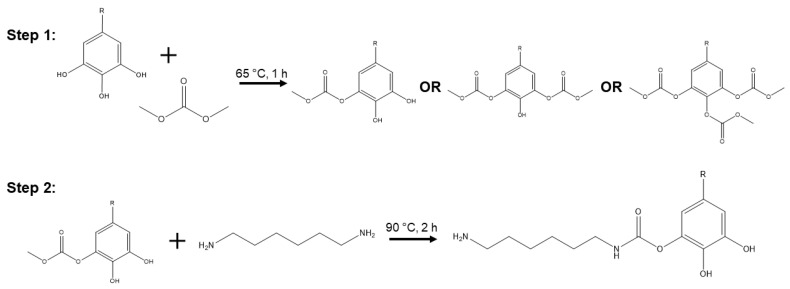
Two-step NIPU synthesis: (**Step 1**) the carbonation of tannic acid with dimethyl carbonate, resulting in mono-, di-, or tri-substitution and (**Step 2**) the formation of a single urethane, shown here with monosubstitution.

The synthesis of non-isocyanate polyurethanes from organic carbonates and tannins or lignin is well established in the literature [14,16,25,26,31]. The two main steps are shown in Figure 1. Hydroxyl groups (in this case, from the gallic acid moieties of tannic acid) undergo transesterification with dimethyl carbonate (DMC), generating methanol and a mixture of substituted carbonates with each degree of substitution [14]. In the next step, hexamethylenediamine reacts with these substituted carbonates to produce urethanes. Alternatively, chitosan can serve as the amine. Recurring combinations result in the NIPU product.

**Figure 2:**
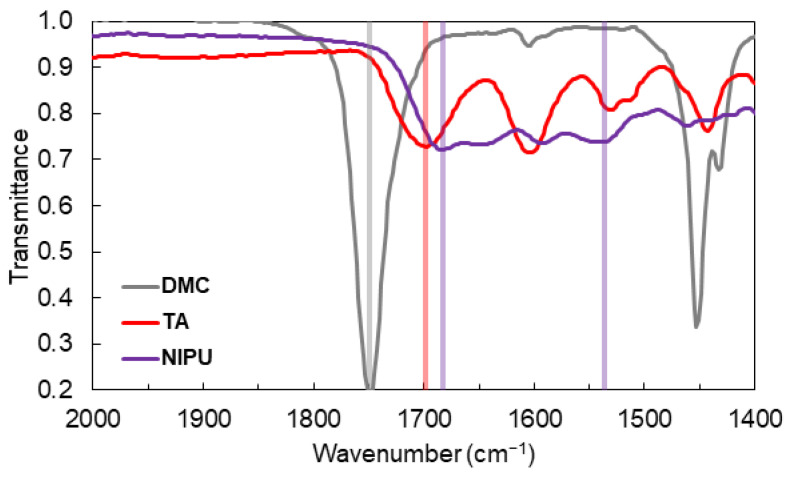
FTIR spectra of substances contributing carbonyl stretches. Lines are added at the maximum absorbance frequency for easier comparison. (DMC: dimethyl carbonate; TA: tannic acid; NIPU: non-isocyanate polyurethane).

To confirm the formation of urethane linkages, ATR-FTIR was performed on the NIPU resin and compared to the spectra of DMC and TA (specifically, the carbonyl stretch), as shown in Figure 2. Dimethyl carbonate exhibits a strong and sharp absorption at 1750 cm^−1^, whereas TA exhibits two carbonyl stretches at 1600 cm^−1^ and 1700 cm^−1^ due to the two different ester positions within the molecule. The electron-withdrawing oxygens in DMC somewhat strengthen the C=O bond, shifting the stretching frequency higher to 1750 cm^−1^. In tannic acid, the ester carbonyl is strengthened by one oxygen but weakened by the resonant ring, which results in a lower energy stretch (1700 cm^−1^). Furthermore, in the formed urethane, the bond is neighbored with two resonant groups: the nitrogen and the aromatic pyrogallol ring. This results in an even lower energy bond near 1680 cm^−1^. A somewhat broad band is also observed at 1537 cm^−1^, characteristic of urethanes [31,33]. There are many overlapping bands in the NIPU spectra, reflecting the various products formed as a result of the reactions between DMC, CH, TA, and hexamethylenediamine. To corroborate FTIR results, ^1^H NMR was performed on the NIPU. Spectra were also obtained for dimethyl carbonate, hexamethylenediamine, chitosan, and tannic acid for comparison (Figure S1). While the complicated structure of possible adducts makes it difficult to assign specific peaks, the NIPU spectrum exhibits unique chemical shifts in the region characteristic of a urethane proton (4.5−9.0 ppm), as shown in Figure S1e.

The final step in NIPU production simultaneously foams and crosslinks the resin. Glutaraldehyde (GA) acts as a crosslinker between the primary amines on the urethane components or chitosan [24,25,26]. The formation of a network increases the viscosity of the NIPU resin, which collapses shortly after foaming in the absence of GA. The self-blowing process is driven by the reaction of carboxylic acid with amines, releasing water [24,25,26]. Observable heat release contributes to exothermic decarboxylation (and the dehydration of the tertiary or secondary alcohols, if present), forming a positive feedback loop. It is expected that acids with more −OH and −COOH groups available for decarboxylation and dehydration produce more porous foams than those with fewer. The properties of foams produced with citric acid, aconitic acid, malic acid, and maleic acid were compared, while maintaining constant molarity.

### 3.2. Foam Structure

**Figure 3:**
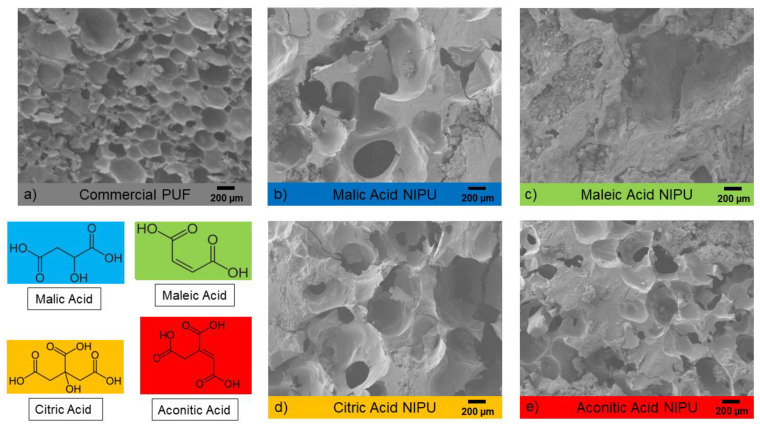
SEM images of each type of polyurethane foam interior.

The SEM images in Figure 3 show the contrast between commercial isocyanate-based PUF and foamed NIPU. Pore size and density varies depending on the acid used, but the overall structure of NIPU foam is more heterogenous than PUF (Figure 3a). Among the NIPU foams, aconitic acid (Figure 3e) appears to produce a structure most similar to PUF, having the highest pore density and the smallest pore size. Maleic acid (Figure 3c) generates a significantly less porous structure, most likely resulting from its lack of hydroxyl and its unsaturated bond. The effect of an additional −COOH is much more significant between maleic acid and aconitic acid than between malic acid and citric acid, which have an extra hydroxyl group that can also contribute to foaming. Carboxyl and hydroxyl groups both appear to influence the foam structure, but their effects do not seem to be independent of each other.

### 3.3. Thermal Properties

**Figure 4:**
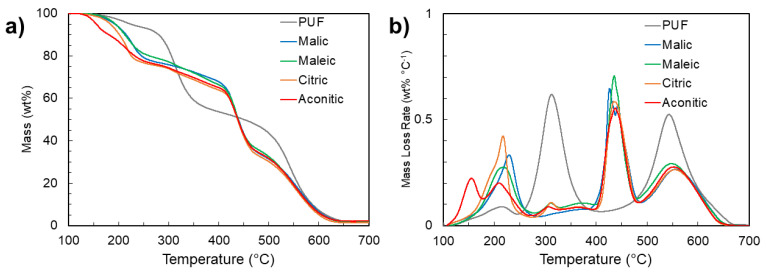
Thermogravimetric analysis (**a**) and derivative thermogravimetric (**b**) curves for PUF and NIPU foams under air.

The thermogravimetric decomposition of each foam sample under air is shown in Figure 4, with key parameters compared in Table 1. Depending on the starting materials, conventional polyurethanes exhibit a two or three-step degradation [22,34,35]. The commercial rigid PUF used here experiences peak mass loss rates at 312 °C and 542 °C. About half of the mass is lost before 400 °C. In contrast, the thermal degradation of each NIPU foam begins as early as 150 °C, depending on the acid used for foaming. This initial degradation is primarily due to the decomposition of each acid molecule (Figure S2a). Regardless of the acid used, about two-thirds of the mass remains at 400 °C. Second and third degradation steps occur afterward, possibly with contributions from chitosan and tannic acid (Figure S2b). Notably, peak mass loss is postponed by over 100 °C in NIPU foams compared to traditional PUF, beginning around 400 °C. The peak thermal degradation temperature of these foams is comparable to that of the lignin-derived NIPU foams made by Sternberg and Pilla [16] and exceeds that of other NIPU foams [17,18,19,21,22].

**Figure 5:**
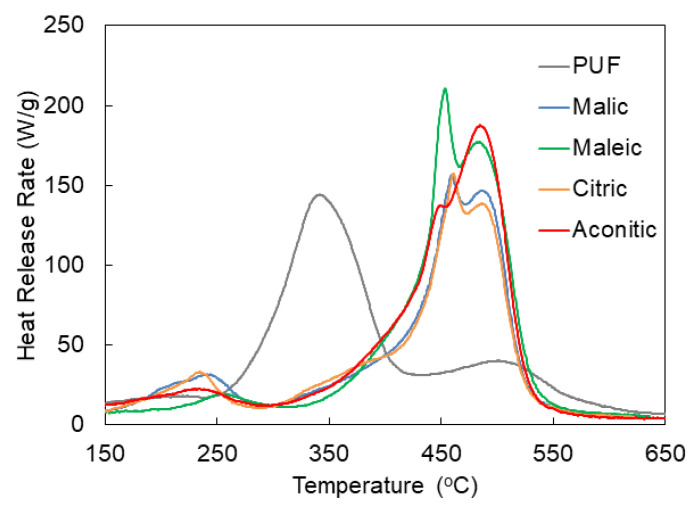
Representative microscale combustion calorimetry results for each foam sample.

To complement oxidative degradation information from TGA, microscale combustion calorimetry (MCC), also known as pyrolysis combustion flow calorimetry (PCFC), was performed on each sample according to method A of ASTM D7309. Representative curves for each sample are plotted in Figure 5. There are clear similarities to the DTG curves (Figure 4b). Each NIPU foam displays minor preliminary heat release, between 200−250 °C, before the peak heat release occurs (between 450 and 485 °C, depending on the acid). Rigid isocyanate-based PUF, on the other hand, experiences peak heat release at 342 °C, with lingering heat release afterward. As in TGA, MCC shows delayed degradation in NIPU foams compared to commercial PU foam. Although the total heat release and peak heat release are similar, major degradation is effectively delayed by >100 °C in NIPU foams (Table 2). The aromatic rings in tannic acid act as radical oxygen scavengers and a char promoter [36].

**Figure 6:**
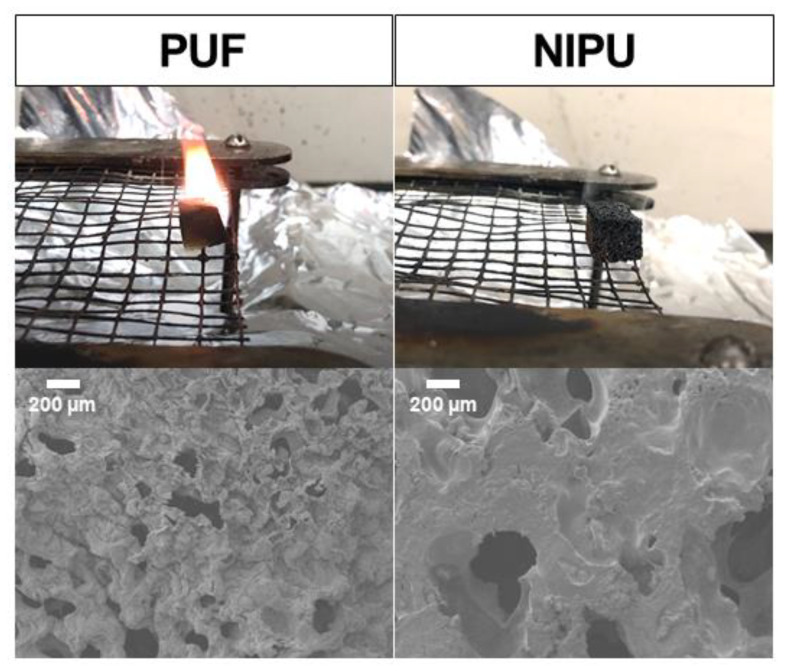
(**Top**) commercial polyurethane foam and NIPU foam 2 s after the removal of a butane torch flame. (**Bottom**) SEM images of both foams’ torched face following torch testing.

While TGA and MCC are useful tools for understanding and predicting the decomposition of materials, they do not accurately represent real fire conditions, and correlations between MCC data and fire tests are not straightforward [37]. To better evaluate flammability, foams were exposed to a butane torch flame for 10 s, with the mass residue and afterburn time measured. These values are reported in Table 3. All the foams (including the traditional PUF) self-extinguish within several seconds, and none exhibit melt dripping (Figure 6). The commercial rigid PUF loses approximately half of its mass during this test, whereas every NIPU foam loses no more than 15%. Except for maleic acid NIPU, the afterburn is also significantly shorter (virtually immediate) for NIPU foams. The afterburn time for maleic acid NIPU is the longest and least consistent, which is not surprising considering its THR and pHRR values are also the highest. Its minimal porosity offers the worst protection from the flame.

TGA and MCC results indicate that major degradation occurs later in NIPU foams, but heat release and total degradation are comparable to commercial PUF. The results of this torch test complement those experiments by demonstrating the resilience of NIPU foams under intense heat. It should be noted that NIPU foam without tannic acid and chitosan burns longer than commercial PUF. Tannic acid contributes to the non-flammability of the foam by acting as a radical scavenger and char source [36], while chitosan contributes as a blowing agent and char source [38]. These natural ingredients render the foam highly resistant to fire by acting in the condensed phase. Post-burn SEM images reveal that the morphology of commercial PUF entirely changes, while NIPU remains largely protected (Figure 6).

## 4. Conclusions

Polyurethanes are versatile materials, but their typical production involves toxic isocyanates. Beyond that, most PUs are flammable and release harsh byproducts. Non-isocyanate polyurethanes can eliminate both disadvantages (flammability and toxicity) with the use of natural components. Chitosan (the second-most abundant organic polymer) and tannic acid (a plant polyphenol)-based NIPU foam exhibits flame resistance and significantly delays thermal degradation compared to traditional PUF. Using benign and renewable reagents in this way to produce a safer foam demonstrates several the Principles of Green Chemistry. Polyurethane composition was confirmed with ^1^H NMR and FTIR spectroscopy. The use of a benign, carboxylic acid−based blowing agent mixture yields a NIPU foam with a pore size relatively comparable to commercial PUF, albeit more heterogenous. The resulting foam is capable of immediately self-extinguishing after a 10 s exposure to a butane torch flame, while maintaining 90% residual mass and pre-burn morphology. Additionally, maximum thermal degradation is postponed by >100 °C compared to traditional foam, as shown with TGA and MCC. The foam described herein represents a single iteration of NIPU incorporating environmentally benign reagents, but it demonstrates more universal potential to replace traditional polyurethanes for insulation and other high-use applications. Any of the components could be replaced by lower cost or more effective chemistries to improve the properties for specific applications.

## Figures and Tables

**Table 1:** Thermogravimetric data for foam samples in oxidative environment.

Sample	T_max_ (°C)	Mass at 400 °C (%)	Mass at 700 °C (%)
PUF	312, 542	53.7	1.5
Malic	229, 426, 556	67.8	1.9
Maleic	218, 435, 548	66.4	1.7
Citric	217, 438, 554	63.9	1.4
Aconitic	155, 209, 437, 553	65.0	2.1

**Table 2:** Microscale combustion calorimetry parameters for each foam sample.

Sample	THR (kJ/g)	pHRR (W/g)	T_pHRR_ (°C)
PUF	16.9 ± 0.1	144.2 ± 3.5	342.5 ± 1.0
Malic	16.4 ± 0.2	160.3 ± 5.5	458.6 ± 4.9
Maleic	18.5 ± 0.5	198 ± 25	456.0 ± 3.2
Citric	15.9 ± 0.7	158.3 ± 7.5	463.5 ± 3.5
Aconitic	17.4 ± 0.9	171 ± 20	485.7 ± 1.8

**Table 3:** Foam flammability results from 10 s butane torch test.

Sample	Mass Residue (%)	Afterburn (s)
PUF	51.4 ± 7.1	4.3 ± 0.7
Malic	88.7 ± 1.6	0.8 ± 0.2
Maleic	89.9 ± 3.1	5.1 ± 5.9
Citric	90.1 ± 0.1	1.0 ± 0.2
Aconitic	84.7 ± 1.6	1.0 ± 0.1

## Data Availability

Data presented in this study are available on request from the corresponding author.

## Supplementary Materials

The following supporting information can be downloaded at: https://www.mdpi.com/article/10.3390/polym14225019/s1, Figure S1: NMR spectrum of dimethyl carbonate (a), hexamethylenediamine (b), chitosan (c), tannic acid (d), and NIPU (e) in DMSO-d6; Figure S2: DTG curves for carboxylic acids (a) and other reagents (b) used in NIPU.
