# Supplementary material for: Non-Isocyanate Polyurethane Bio-Foam with Inherent Heat and Fire Resistance
Source: Polymers (Basel). 2022 Nov 19;14(22):5019. doi: 10.3390/polym14225019 (PMC9697988; doi:10.3390/polym14225019)
Supplement: Supplementary file 1 [file polymers-14-05019-s001.zip › polymers-1954855-supplementary.pdf]

Supporting Information

Non-Isocyanate Polyurethane Bio-Foam with Inherent Heat and Fire Resistance

Dallin L. Smith,[a] Danixa Rodriguez-Melendez,[a] Sidney M. Cotton,[a] Yufeng Quan,[b] Qingsheng Wang,[b] and Jaime C. Grunlan[a,c,d]*

[a] Department of Chemistry, Texas A&M University, College Station, TX 77843

[b] Department of Chemical Engineering, Texas A&M University, College Station, TX 77843

[c] Department of Mechanical Engineering, Texas A&M University, College Station, TX 77843

[d] Department of Materials Science and Engineering, Texas A&M University, College Station, TX 77843

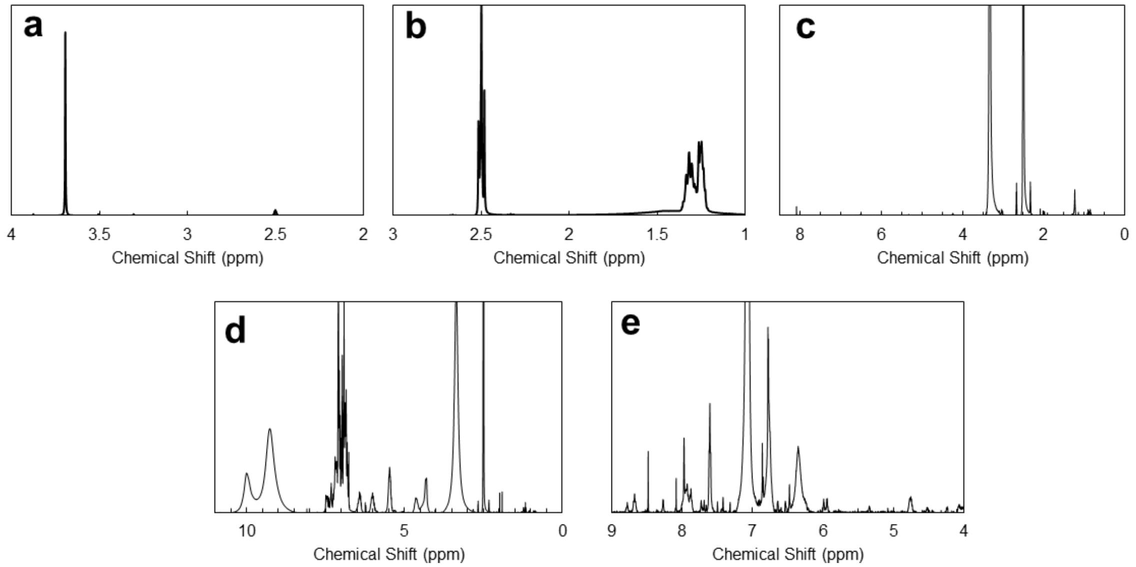

Figure S1. NMR spectrum of dimethyl carbonate **(a)**, hexamethylenediamine **(b)**, chitosan **(c)**, tannic acid **(d)**, and NIPU **(e)** in DMSO-d6.

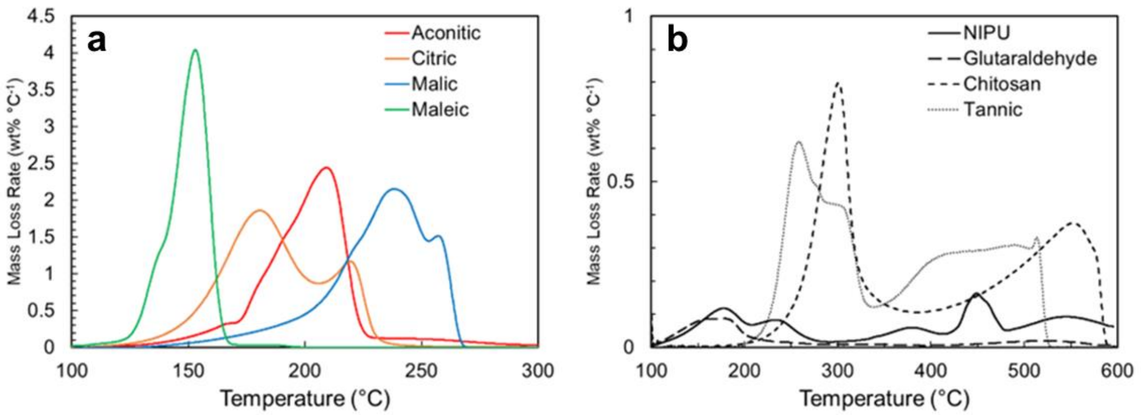

Figure S2. DTG curves for carboxylic acids **(a)** and other reagents **(b)** used in NIPU.
